# A digitally supported guided self-help for depression and anxiety –The development and results of the Finnish guided self-help (F-GSH)

**DOI:** 10.1192/j.eurpsy.2025.597

**Published:** 2025-08-26

**Authors:** S. E. Saarni, K. Mikkonen, P. Roslund, S. Laitala, S. I. Saarni

**Affiliations:** 1 Psychiatry, HUS, Helsinki; 2 Psychiatry, University of Tampere, Tampere; 3 Päijät-Häme Well Being County, Lahti; 4 Helsinki University; 5 HUS, Helsinki, Finland

## Abstract

**Introduction:**

*Increasing access to short, evidence-based psychotherapies is essential for responding to the rapid increase of demand. This can be achieved only by implementing scalable digital solutions. The Finnish guided self-help (F-GSH) is a digitally supported treatment based on Cognitive Behavioral Therapy (CBT). It was implemented in 2020 onward as part of the national First Line Therapies initiative to face the increased demand and shortage of therapists in primary health care.*

**Objectives:**

*We describe the implementation and preliminary results of the Finnish Guided Self-Help (F-GSH) of GSH for depression and anxiety in adults.*

**Methods:**

*Description of the digitally supported F-GSH and therapist training using e-learning platform. We report preliminary outcomes from F-GSH for depression (n=766) and anxiety (n=1043). As outcomes we report self-reported depression (PHQ-9) and anxiety (GAD-7) symptoms measured at the beginning and end of the F- GSH treatment, patient and employee satisfaction, and proportion of patients in need of further treatment after F-GSH. Results are gathered from a wide geopraphical area covering 12 well-being counties.*

**Results:**

*The training program includes a 5-7 hours online training. A multiple-choice exam must be passed at the end course to gain certification. The training can be enhanced locally by skills workshops, to refine abilities, discuss specific GSH programs and patient groups, or address local implementation issues. In August 2024 more than 2700 employees had undergone the F-GSH training. The symptom severity changes during the treatment in our sample were as follows: Depression program the mean PHQ-9 for depression were at the beginning and end of the treatment 15.4 (SD 5.4)- 11.2(6.0) and for anxiety symptoms GAD-7 10.3(4.7)-7.9(4.8). Respectively, for during the treatment program targeted for anxiety symptoms the GAD-7 levels were 12.25(4.8)- 8.8(5.0) and for PHQ-9 12.43(5.5)- 9.1(5.6). Worsening of the symptoms were reported for 0-2% of the patients. Further treatment needed 38% of patients after F-GSH for depression and After F-GSH 38% of patients had no need for any further treatment.*

**Conclusions:**

*E-learning platform forms a scalable and acceptable solution for training large number of professionals on a short time period at low cost for an evidence based treatment model. The symptoms reductions during the digitally supported F-GSH for depression and anxiety were comparable with previously reported in other studies, and comparable with longer CBT treatments. The F-GSH seems to be acceptable for the patients and employees. Also, the symptoms seem to ameliorate clinically significantly during the treatment.*

**Disclosure of Interest:**

None Declared

